# Qualitative and quantitative assessment of sperm miRNAs identifies hsa-miR-9-3p, hsa-miR-30b-5p and hsa-miR-122-5p as potential biomarkers of male infertility and sperm quality

**DOI:** 10.1186/s12958-022-00990-7

**Published:** 2022-08-15

**Authors:** Meghali Joshi, Syed Waseem Andrabi, Ranjeet Kumar Yadav, Satya Narayan Sankhwar, Gopal Gupta, Singh Rajender

**Affiliations:** 1 Division of Endocrinology, Central Drug Research Institute, Lucknow, India; 2 Department of Zoology, Lucknow University, Lucknow, India; 3 King George’s Medical University (KGMU), Lucknow, India; 4 Academy of Scientific and Innovative Research (AcSIR), Ghaziabad, India

**Keywords:** microRNA, Male infertility, Sperm miRNAs, Sperm quality biomarkers, Spermatogenesis

## Abstract

**Background:**

In contrast with the preceding stages of the germ cells, spermatozoa are unusually rich in small non-coding RNAs in comparison to the coding RNAs. These small RNAs may have had an essential role in the process of spermatogenesis or may have critical roles in the post-fertilization development. Sporadic efforts have identified a few differentially expressed miRNAs in infertile individuals, which do not replicate in other studies.

**Methods:**

In order to identify miRNAs signatures of infertility or poor sperm quality, we compared miRNA differential expression data across nine datasets, followed by their analysis by real-time PCR in a case–control study. This was followed by the validation of potential biomarkers in yet another set of cases and controls. For this, total RNA was isolated from 161 sperm samples. miRNA expression levels in infertile cases and fertile controls were measured using TaqMan real-time PCR. Meta-analyses of two miRNAs (hsa-miR-9-3p and hsa-miR-122-5p) were performed using Comprehensive Meta‐Analysis Software (version 2). All statistical analyses were performed with the help of GraphPad Prism Software (version 8).

**Results:**

Literature search identified seven miRNAs (hsa-let-7a-5p, hsa-miR-9-3p, hsa-miR-22-5p, has-miR-30b-5p, hsa-miR-103-3p, hsa-miR-122-5p and hsa-miR-335-5p) showing consistent dysregulation in infertility across a minimum of four studies. In the discovery phase, six miRNAs showed strong association with infertility with four (hsa-miR-9-3p, hsa-miR-30b-5p, hsa-miR-103-3p and hsa-miR-122-5p) showing consistent differential regulation across all sub-groups. Receiver operating characteristic (ROC) curve analysis showed that the area under curve of > 0.75 was achieved by three (hsa-mir-9-3p, hsa-miR-30b-5p and hsa-miR-122-5p) miRNAs. In the validation phase, these three miRNAs showed consistent association with infertility (hsa-mir-9-3p, hsa-miR-30b-5p, and hsa-miR-122-5p). Meta-analysis on hsa-miR-122-5p showed its significant quantitative association with infertility [Hedge’s g = -2.428, *p* = 0.001 (Random effects)].

**Conclusions:**

Three miRNAs (hsa-miR-9-3p, hsa-miR-30b-5p and hsa-miR-122-5p) have strong linkage with infertility and a high potential as sperm quality biomarkers.

**Supplementary Information:**

The online version contains supplementary material available at 10.1186/s12958-022-00990-7.

## Background

Normozoospermia is not synonymous with fertility, raising concerns about altered molecular milieu in sperm as a cause of infertility in such individuals [1, 2]. Similarly, in oligozoospermic infertile individuals, infertility may be because of factors beyond a mere drop in sperm count [3]. In a high number of assisted reproduction trials, post-fertilization development does not ensue, resulting in the cessation of development at various levels pre- or post-conception [4, 5]. This raises concerns about sperm quality, which are not covered under the traditional semen analysis parameters [6]. Therefore, identification of sperm quality biomarkers has value in assessing semen samples for quality and fertility, which can be a boon for assisted reproduction [7]. Currently, the advanced laboratory tests such as anti-sperm antibody test, hemizona test, quantification of reactive oxygen species (ROS), sperm DNA fragmentation assessment are used as the second line of tests in infertility evaluation [8]. A male gamete is not just a vehicle that delivers the male genetic material to the oocyte; additionally, a sperm provides a highly structured genome with specific epigenetic marks in the form of DNA modifications [9, 10] and a plethora of RNA molecules [11]. Further, DNA methylation alterations [9, 10, 12] and sperm protein changes [13] have also been shown to affect fertility and post-fertilization development. Therefore, sperm quality analysis at present ignores a complex molecular milieu, the investigation of which could aid in the identification of sperm quality markers for use in assisted reproduction.

Spermatozoa are unusually rich in small non-coding RNAs [14], which could have played an essential role in the process of spermatogenesis or may have critical roles in the post-fertilization development [15]. Nixon et al., (2015) identified that spermatozoa get loaded with diverse miRNAs during the epididymal maturation, suggesting their roles in the post-fertilization development [16]. Sperm obtained from Drosha and Dicer germ-line specific conditional knockout mice showed reduced developmental potential of the embryos, which were rescued by injecting sncRNAs derived from the wild-type spermatozoa into the embryos, suggesting that sncRNAs are crucial for the development of pre-implantation embryos [17]. Sperm-borne miRNAs, such as miR-34c have been shown to be critical for the first cleavage in the zygote [18]. Hua et al. 2019 identified five miRNAs (miR-132-3p, miR-191-3p, miR-520a-5p, miR-101-3p and miR-29a-3p) that could serve as potential markers for sperm quality assessment in IVF [7]. In another study, the authors reported that sperm with high miR-191-5p expression had higher fertilization rate (FR), effective embryo rate (EER) and high quality embryo rate (HQER), suggesting that miR-191-5p could be used as a potential biomarker to detect high quality sperm for in-vitro fertilization [19]. Sperm miRNAs have also gained attention due to their emerging roles in transgenerational inheritance of a number of acquired characters and their influence on a number of health conditions [20, 21].

In the first ever attempt to identify the potential biomarkers of sperm fertility/quality, Abu-Halima et al. (2014) selected five miRNAs (hsa-miR-34b*, hsa-miR-34b, hsa-miR-34c-5p, hsa-miR-122 and hsa-miR-429) and suggested their potential to distinguish sub-fertile group from the fertile group [22]. In another report, Vazquez et al. (2019) selected the best miRNA pairs from their previously published data and showed that the hsa-miR-942-5p/hsa-miR-1208 pair displayed the best potential for the diagnosis of infertile men with seminal alterations while the hsa-miR-34b-3p/hsa-miR-93-3p pair showed the best potential for the diagnosis of infertile men with unexplained male infertility or when the seminal parameters were close to the threshold values [2]. Till date, a few studies on fertile and infertile sperm samples have identified hundreds of differentially expressed miRNAs [23–30]. A number of these differentially expressed miRNAs are shared across these studies, offering the opportunity to identify the miRNA-based markers of sperm quality. In order to identify the miRNAs signatures of infertility or poor sperm quality, we compared miRNA differential expression data across nine datasets, followed by the analysis of strong candidate miRNAs in an independent set of infertile cases and fertile controls.

## Materials and methods

### Literature search

In order to identify the most promising miRNAs for in-depth analysis, we performed a systematic review of the literature using PubMed, Google Scholar and ResearchGate databases. The search terms included sperm miRNA, male infertility, oligozoospermia, asthenozoospermia, teratozoospermia, normozoospermia, and infertility in various combinations. The miRNA expression data were collected from previously published articles. Eight studies (two microarray and six RT-PCR based) had investigated the expression of miRNAs in sperm/semen of infertile patients till date (Table 1). We also included our miRNA sequencing data for oligo/oligoasthenozospermia infertile Indian patients [31]. Thus, nine datasets were compared to identify common differentially expressed miRNAs in male infertility.

**Table 1:** Summary of miRNA profiling studies in human sperm

Study	Technique used	Sample type	Sample size	Cut off value	No of miRNAs analysed
Liu et al. 2012 [23]	Microarray	Infertile and Normozoospermic fertile (NF) (semen)	86 infertile and 86 NF	> 1.5 or < 0.66	2844 miRNAs
Halima et al. 2013 [24]	Microarray	Oligoasthenozoospermia (OA), A (asthenozoospermia) and NF (sperm)	9 OA, 9A and 9 NF	> 1.5 or < 0.66	1205 miRNAs
Abhari et al. 2014 [25]	qRT-PCR	Oligozoospermia (O) and NF (sperm)	43 O and 43 NF	-	2 miRNAs
Salas-Huetos et al. 2015 [26]	qRT-PCR	O, A, teretozoospermia (T) and NF (sperm)	10 O, 10 A, 10 T and 10 NF	-	736 miRNAs
Muñoz et al. 2015 [27]	qRT-PCR	O and NF (sperm)	9 O and 7 NF	-	23 miRNAs
Salas-Huetos et al. 2016 [28]	qRT-PCR	Normozoospermic infertile (NI) and NF (sperm)	8 NI and 8NF	-	736 miRNAs
Huan et al. 2017 [29]	qRT-PCR	O, A, OA and NF (semen)	36 O, 36 A, 36 OA and 36 NF	-	6 miRNAs
Mokánszki et al. 2020 [30]	qRT-PCR	O, A and NF (sperm)	10 O, 10 A and 10 NF	-	11 miRNAs

### Case–control study

The study was approved by the Institutional Review Board and Ethics Committee of the Central Drug Research Institute (CDRI), Lucknow. A written informed consent was obtained from all the participants before enrollment. Semen samples were collected from the male partners of couples undergoing assisted reproduction for infertility treatment. Female factor infertility in these couples had been ruled out on the basis of standard clinical and laboratory evaluation of the female partners. The length of infertility in these individuals ranged from 2 to 10 years. Individuals with Y-chromosome microdeletions or those with any history of surgery or genital tract obstruction, cryptorchidism and vasectomy were excluded from this study. Age-matched normozoospermic fertile men having fathered a child within the last two years without assisted reproduction were enrolled as controls. Following the above, a total of 161 semen samples were collected and classified into: oligo/oligoasthenozoospermic infertile (*n* = 41), asthenozoospermic infertile (*n* = 40), normozoospermic infertile (*n *= 40) and normozoospermic fertile (*n* = 40). Half the number of samples in each group were used in the discovery phase and half in the validation phase.

**Fig. 1:**
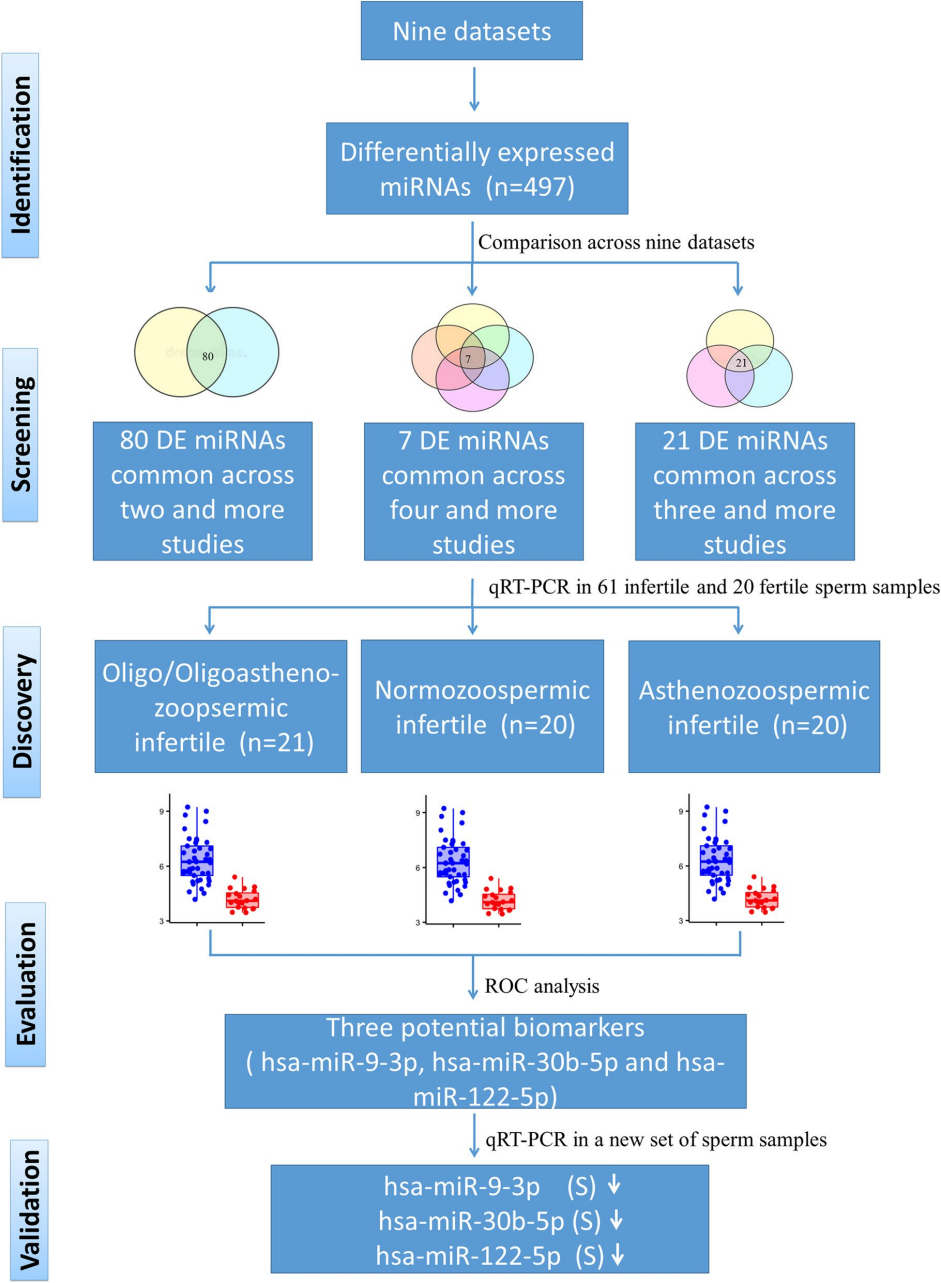
Schematic flow chart of the study design. This study was divided in three phases; screening from literature, discovery and evaluation of infertility markers, and validation of the potential biomarkers

Semen samples were obtained from the participants by masturbation and collected in sterile containers after 3 days of sexual abstinence. After liquefaction, semen samples were examined for sperm concentration, motility and morphology as per the WHO 2010 criteria [32]. The characteristics of the enrolled cases and age-matched controls are presented in Table 2. In order to remove somatic cells present in the ejaculate, semen samples were processed according to the somatic cell lysis method [33]. To further ensure the complete removal of somatic cells, conventional PCR was performed for markers, such as CD45 (leucocyte specific), PRM1 (sperm specific) and KIT (immature germ cells specific). The overall study was conducted as presented in the schematic Fig. 1.

**Table 2:** Semen parameters of normozoospermic fertile controls (NF) and infertile patients: oligo/oligoastheno-zoospermic (O/OA), normozoospermic infertile (NI) and asthenozoospermic (A)

**Discovery Phase**				
	**NF**	**O/OA**	**NI**	**A**
Average sperm concentration (million/ml) ± SD	91.95 ± 38.12	7.19 ± 3.17***	74.2 ± 34.86 ^ns^	49.2 ± 33.19***
Average sperm motility (%) ± SD	51.7 ± 10.77	20.19 ± 14.75***	51.35 ± 10.20 ^ns^	17.7 ± 4.12***
Average Age (years) ± SD	32.75 ± 3.97	33.19 ± 5.24	33.65 ± 3.42	33.85 ± 3.42
**Validation Phase**				
	**NF**	**O/OA**	**NI**	**A**
Average sperm concentration (million/ml) ± SD	71.29 ± 28.33	6.45 ± 3.48***	59 ± 27.05^ ns^	45.75 ± 27.54***
Average sperm motility (%) ± SD	50.20 ± 8.90	16.91 ± 10.16***	42.5 ± 8.17*	18.5 ± 4.89***
Average Age (years) ± SD	33.55 ± 3.26	33.2 ± 4.42	34.65 ± 4.18	35.95 ± 3.63

^ns^*p* > 0.05,*p < 0.05, **p < 0.005, ***p < 0.0005

### Total RNA isolation and quantification

Total RNA, including miRNAs were isolated from sperm samples using MasterPure Complete DNA & RNA Purification Kit (Epicentre) according to the protocol recommended by the manufacturers. Total RNA was quantified using Qubit RNA HS Assay Kit (Invitrogen, USA) and measured by Qubit 2.0 fluorometer (Life Technologies, USA).

### Reverse transcription and quantitative Real-Time PCR

50 ng of total RNA per 15 µl reaction was reverse transcribed into single-stranded cDNA using the TaqMan microRNA reverse transcription kit according to the protocol recommended by the manufacturers. Relative quantitative real-time PCR was performed on an ABI StepOne Plus Real-Time PCR System (Applied Biosystems) using Maxima Probe/ROX qPCR Master Mix (ThermoFisher Scientific, USA) for all TaqMan MicroRNA assays for hsa-let-7a-5p, hsa-miR-9-3p, hsa-miR-22-5p and hsa-miR-30b-5p, hsa-miR-103a-3p, hsa-miR-122-5p, and hsa-miR-335-5p. The RNU6B snRNA TaqMan microRNA assay (ThermoFisher Scientific, USA) was chosen as an endogenous reference for normalization. All reactions were prepared according to the manufacturers’ recommendations and carried out in triplicates.

### Statistical analysis

Statistical analyses were performed with the help of GraphPad Prism Software version 8 (GraphPad Software, San Diego, CA, USA). Non-parametric Mann Whitney U test was used to evaluate the differences in miRNA expression levels between cases and controls. Spearman’s correlation was used to measure the association between sperm parameters (sperm concentration, motility) with the normalized Ct value of each miRNAs. *P* < 0.05 was considered to be statistically significant. In order to identify the potential of selected miRNAs to discriminate infertile from fertile samples, area under ROC curve (AUC) were obtained. AUC of 0.75 was taken to be good for a biomarker. The power of statistical comparison of expression between cases and controls was 100%, suggesting adequate sample size for this study.

### Quantitative analysis

Additionally, for quantitative analysis, we performed meta-analyses on the miRNAs where quantitative data for more than three studies/datasets were available. The above studies were further subjected to well-defined inclusion/exclusion criteria. Inclusion criteria for meta-analyses were as follows; a) studies that had analysed the miRNA expression in human sperm, b) inclusion of the patients was done according to the standard diagnostic parameters, c) the purpose of all the studies was similar, d) sufficient data and information were provided for meta-analysis. Review articles, studies that failed to provide detailed description of the subjects, data and other information to precisely understand the study design and data therein, were excluded. The unavailability of sufficient quantitative data restricted our meta-analysis to only two miRNAs (hsa-miR-122-5p and hsa-miR-9-3p). Comprehensive Meta‐Analysis Software (version 2) was used to perform all statistical analyses. Hedge’s g was used as the ‘effect size’ with their respective 95% confidence intervals. The heterogeneity between the studies was quantitatively assessed using I^2^ statistics. *P *value of < 0.10 was considered statistically significant due to low power of the heterogeneity test. I^2^ value was used to determine the magnitude of heterogeneity, where I^2^ value of < 25% means low, < 50% means medium and < 75% means high heterogeneity [34]. Publication bias was evaluated using the Egger’s regression test of significance.

### Target prediction and functional analysis

A search in the online database miRWalk version 2.0 was used to identify the targets of hsa-miR-9-3p, hsa-miR-30b-5p and hsa-miR-122-5p. We selected the species ‘Human’, database(s) ‘miRBase’, and identifier types ‘MiRNA’ and searched for one miRNA at a time. Further, we chose four algorithms (miRWalk, miRanda, TargetScan, and miRDB) to obtain common miRNA targets. Gene ontology and pathway analyses were done using online database ‘Panther’.

## Results

### Selection of consistently differentially expressed miRNAs

**Fig. 2:**
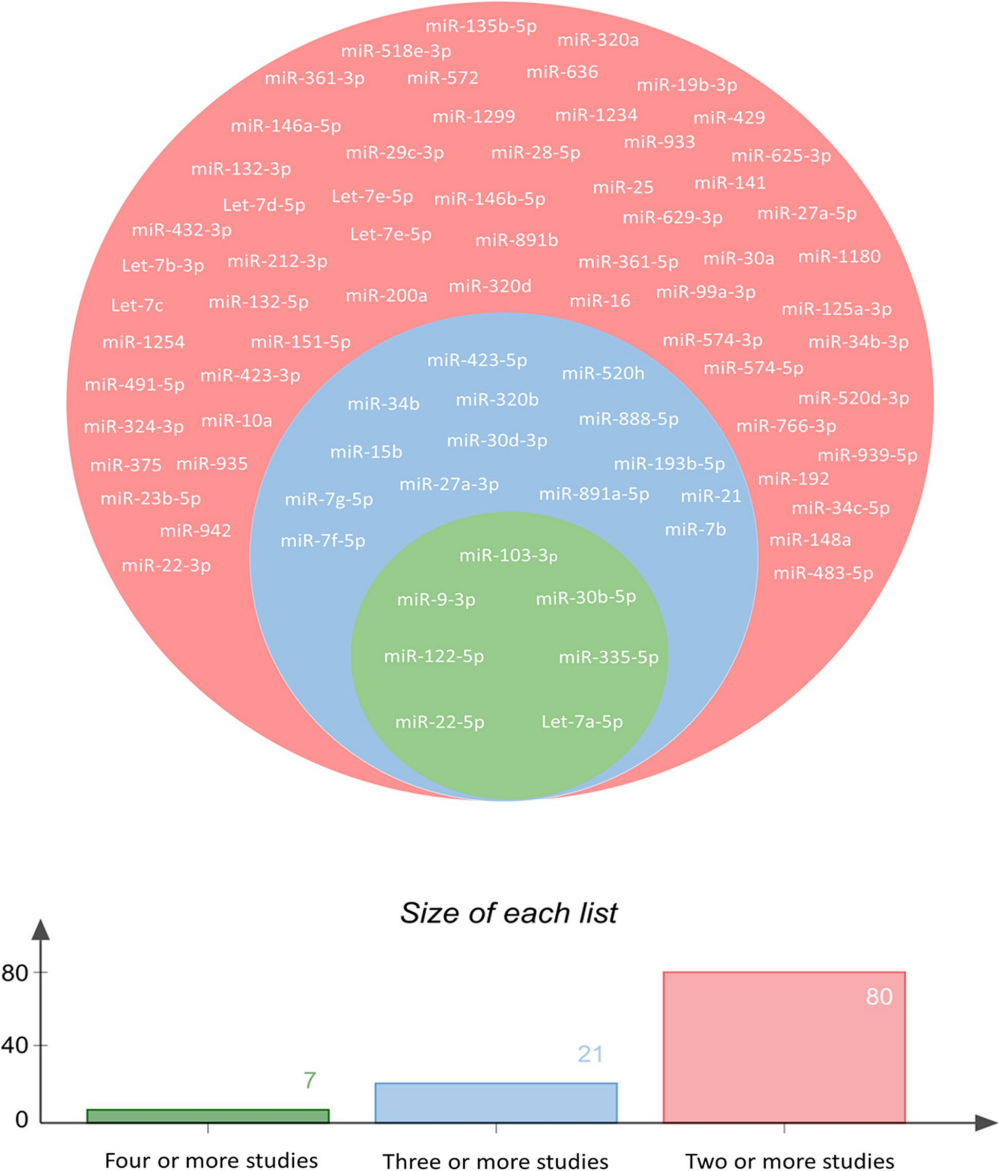
Venn diagram showing the common and unique differentially expressed miRNAs across nine data-sets. First circle (green) shows differentially expressed miRNAs common across four or more studies, second circle (blue) shows differentially expressed miRNAs common across three or more studies and third circle (red) shows differentially expressed miRNAs common across two or more studies

miRNA data from nine data-sets were compared to identify the most commonly differentially expressed miRNAs. Seven studies had conducted miRNA profiling in oligozoospermic patients, five in asthenozoospermic patients and one each in normozoospermic and teratozoospermic infertile patients. A total of 497 miRNAs were found to be differentially expressed across nine data-sets. Upon comparing the miRNAs reported in nine studies, we found that seven miRNAs were differentially expressed in four or more studies, twenty-one miRNAs were differentially expressed in three or more studies and eighty miRNAs were differentially expressed in two or more studies (Fig. 2). Considering differential expression of seven miRNAs in the highest number of studies, there is a strong likelihood that they are linked to the pathogenesis of male infertility and their differential consistent expression across studies suggests their potential as biomarkers of sperm quality. These seven miRNAs (hsa-let-7a-5p, hsa-miR-9-3p, hsa-miR-22-5p, hsa-miR-30b-5p, hsa-miR-103-3p, hsa-miR-122-5p and hsa-miR-335-5p) were selected for investigation as potential biomarkers of fertility/sperm quality.

### Discovery of potential infertility markers

**Fig. 3:**
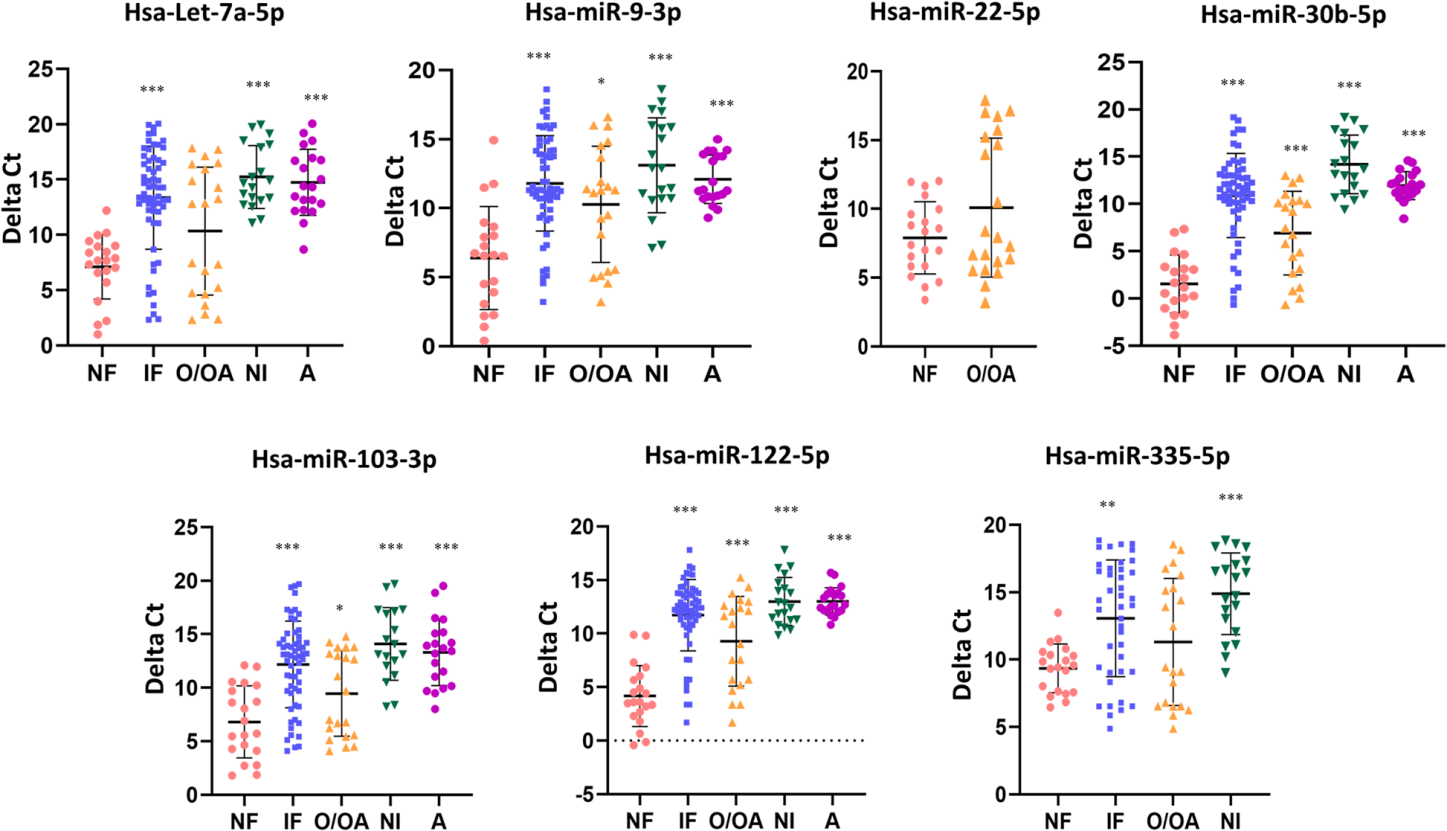
Abundance levels of seven selected miRNAs in cases and controls. The abundance levels were analysed in three infertile categories, oligo/oligoastheno-zoospermia, normozoospermic infertile, asthenozoospermia. Data are presented as ∆Ct (lower ∆Ct = higher abundance level). Non-parametric Mann–Whitney U test was used to evaluate differences in the abundance levels. Significant differences from the normozoospermic fertile control are indicated: *p < 0.05, **p < 0.005, ***p < 0.0005. NF, IF, O/OA, NI, A indicate normozoospermic fertile, infertile, oligo/oligoasthenozoospermic, normozoospermic infertile, asthenozoospermic groups, respectively

All seven selected miRNAs were analysed in a set of oligo/oligoasthenozoospermic infertile (*N* = 21), asthenozoospermic infertile (*N* = 20) normozoospermic infertile (*N* = 20), and normozoospermic fertile (*N* = 20) individuals. We found that six miRNAs (hsa-let-7a-5p, hsa-miR-9-3p, hsa-miR-30b-5p, hsa-miR-103-3p, hsa-miR-122-5p and hsa-miR-335-5p) were significantly down-regulated in infertile samples as compared to fertile samples. The mean abundance level was 0.01 –folds for hsa-let-7a-5p, 0.02 –folds for hsa-miR-9-3p, 0.01 –folds for hsa-miR-30b-5p, 0.02 –folds for hsa-miR-103-3p, 0.05 –folds for hsa-miR-122-5p and 0.07 –folds for hsa-miR-335-5p in infertile samples as compared to fertile samples (Fig. 3). Statistical comparison using Mann Whitney U test showed significantly reduced expressions of hsa-let-7a-5p (cases median ∆Ct 13.99, controls median ∆Ct 7.67), hsa-miR-9-3p (cases median ∆Ct 11.35, controls median ∆Ct 6.58), hsa-miR-30b-5p (cases median ∆Ct 11.43, controls median ∆Ct 1.41), hsa-miR-103-3p (cases median ∆Ct 12.98, controls median ∆Ct 6.31), hsa-miR-122-5p (cases median ∆Ct 12.38, controls median ∆Ct 3.6) and hsa-miR-335-5p (cases median ∆Ct 14.03, controls median ∆Ct 9.50) in the cases in comparison to the controls.

We also compared their expression across infertility sub-groups. In the oligo/oligoasthenozoospermic group, five miRNAs were down-regulated (hsa-let-7a-5p, hsa-miR-9-3p, hsa-miR-30b-5p, hsa- miR-103-3p and hsa-miR-122-5p) in comparison to the normozoospermic fertile group, of which four (hsa-miR-9-3p, hsa-miR-30b-5p, hsa-miR-103-3p and hsa-miR-122-5p) reached statistical significance (Fig. 3). The mean abundance level was 0.13 –folds for hsa-miR-9-3p, 0.10 –folds for hsa-miR-30b-5p, 0.23 –folds for hsa-miR-103-3p and 0.13 –folds for hsa-miR-122-5p in oligo/oligoasthenozoospermic samples as compared to normozoospermic fertile samples (Fig. 3). Statistical comparison using Mann Whitney U test showed significantly reduced expressions of hsa-miR-9-3p (cases median ∆Ct 11.29, controls median ∆Ct 6.58), hsa-miR-30b-5p (cases median ∆Ct 7.45, controls median ∆Ct 1.41), hsa-miR-103-3p (cases median ∆Ct 9.71, controls median ∆Ct 6.31) and hsa-miR-122-5p (cases median ∆Ct 10.89, controls median ∆Ct 3.60) in the cases in comparison to the controls, while hsa-let-7a-5p expression in the cases was reduced (cases median ∆Ct 12.83, controls median ∆Ct 7.67), the difference was not statistically significant. The expressions of hsa-miR-22-5p (cases median ∆Ct 7.91, controls median ∆Ct 7.86) and hsa-miR-335-5p (cases median ∆Ct 9.42, controls median ∆Ct 9.50) were comparable between cases and controls.

In the normozoospermic infertile group, six (hsa-let-7a-5p, hsa-miR-9-3p, hsa-miR-30b-5p, hsa-miR-103-3p, hsa-miR-122-5p and hsa-miR-335-5p) out of seven miRNAs were significantly down-regulated as compared to the normozoospermic fertile group. The mean abundance level was 0.001 –folds for hsa-let-7a-5p, 0.009 –folds for hsa- miR-9-3p, 0.0001 –folds for hsa-miR-30b-5p, 0.008 –folds for hsa-miR-103-3p, 0.001 –folds for hsa-miR-122-5p and 0.003 –folds for hsa-miR-335-5p in normozoospermic infertile samples as compared to normozoospermic fertile samples (Fig. 3). Statistical comparison using Mann Whitney U test showed significantly reduced expressions of hsa-let-7a-5p (cases median ∆Ct 14.68, controls median ∆Ct 7.67), hsa-miR-9-3p (cases median ∆Ct 12.90, controls median ∆Ct 6.58), hsa-miR-30b-5p (cases median ∆Ct 13.68, controls median ∆Ct 1.41), hsa-miR-103-3p (cases median ∆Ct 13.60, controls median ∆Ct 6.31), hsa-miR-122-5p (cases median ∆Ct 12.62, controls median ∆Ct 3.6) and hsa-miR-335-5p (cases median ∆Ct 15.42, controls median ∆Ct 9.50) in the cases in comparison to the controls.

In the asthenozoospermic infertile group, five miRNAs (hsa-let-7a-5p, hsa-miR-9-3p, hsa-miR-30b-5p, hsa-miR-103-3p, and hsa-miR-122-5p) were significantly down-regulated in comparison to the normozoospermic fertile samples. The mean abundance was 0.004 –folds for hsa-let-7a-5p and hsa-miR-9-3p, 0.0002 –folds for hsa-miR-30b-5p, 0.01 –folds for hsa-miR-103-3p and 0.0007 –folds for hsa-miR-122-5p in asthenozoospermic samples as compared to normozoospermic fertile samples (Fig. 3). The expression of hsa-miR-22-5p was below the detection level in asthenozoospermic and normozoospermic infertile samples, and hsa-miR-335-5p was below the detection level in asthenozoospermic samples. Statistical comparison using Mann Whitney U test showed significantly reduced expressions of hsa-let-7a-5p (cases median ∆Ct 13.99, controls median ∆Ct 7.67), hsa-miR-9-3p (cases median ∆Ct 11.35, controls median ∆Ct 6.58), hsa-miR-30b-5p (cases median ∆Ct 11.43, controls median ∆Ct 1.41), hsa-miR-103-3p (cases median ∆Ct 12.98, controls median ∆Ct 6.31) and hsa-miR-122-5p (cases median ∆Ct 12.66, controls median ∆Ct 3.6) in the cases in comparison to the controls.

Thus, the above results suggest that four (hsa-miR-9-3p, hsa-miR-30b-5p, hsa-miR-103-3p, and hsa-miR-122-5p) out of seven miRNAs were consistently differentially expressed in the three categories of infertile patients, whereas four (hsa-miR-9-3p, hsa-miR-30b-5p, hsa-miR-103-3p and hsa-miR-122-5p), six (hsa-let-7a-5p, hsa-miR-9-3p, hsa-miR-30b-5p, hsa-miR-103-3p, hsa-miR-122-5p and hsa-miR-335-5p) and five (hsa-let-7a-5p, hsa-miR-9-3p, hsa-miR-30b-5p, hsa-miR-103-3p and hsa-miR-122-5p) miRNAs were differentially expressed in oligo/oligoasthenozoospermic, normozoospermic infertile and asthenozoospermic infertile samples, respectively.

### Correlation of the miRNA abundance level (∆Ct) and semen parameters (sperm concentration and motility)

The correlations of the abundance of seven miRNAs (hsa-let-7a-5p, hsa-miR-9-3p, hsa-miR-22-5p, hsa-miR-30b-5p, hsa-miR-103-3p, hsa-miR-122-5p and hsa-miR-335-5p) with sperm concentration and the abundance of five miRNAs (hsa-let-7a-5p, hsa-miR-9-3p, hsa-miR-30b-5p, hsa-miR-103-3p and hsa-miR-122-5p) with sperm motility were assessed. The results showed that the abundance levels of three miRNAs i.e. hsa-miR-9-3p, hsa-miR-30b-5p and hsa-miR-122-5p were significantly positively correlated with sperm concentration (correlation coefficients (r) with ∆Ct were -0.312, -0.450 and -0.438, respectively) (Supplementary Table 1). The lower the abundance levels of the miRNAs (i.e. higher ∆Ct), the lower was sperm count. Previously, the expression of hsa-miR-122-5p was significantly positively correlated with the concentration of sperm in semen [34]. Further, all five miRNAs (hsa-let-7a-5p, hsa-miR-9-3p, hsa-miR-30b-5p, hsa-miR-103-3p and hsa-miR-122-5p) showed significant positive correlation with sperm motility with correlation coefficients (r) of -0.786, -0.736, -0.771, -0.559 and -0.795 for hsa-let-7a-5p, hsa-miR-9-3p, hsa-miR-30b-5p, hsa-miR-103-3p and hsa-miR-122-5p, respectively. The lower the abundance levels of the miRNAs (i.e. higher ∆Ct), the lower was sperm motility (Supplementary Table 1).

### Evaluation of potential sperm quality markers

**Fig. 4:**
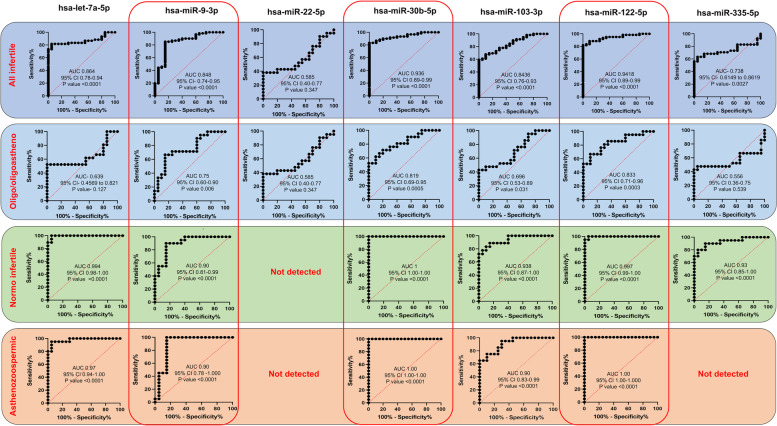
Receiver operating characteristic (ROC) curves of seven selected miRNAs to determine their ability to discriminate between infertile and fertile samples. ROC curve analysis was performed in infertile and in three infertile categories: oligo/oligoastheno-zoospermia, normozoospermic infertile and asthenozoospermia. CI indicates confidence interval. In normozoospermic infertile individuals, miR-22-5p was not detected, and in asthenozoospermic infertile individuals, miR-22-5p and miR-335-5p were not detected

Receiver operating characteristic (ROC) curve analysis was conducted to assess the potential of each miRNAs as a biomarker of sperm quality. Area under curve was performed for six miRNAs in infertile patients. The highest AUC score (0.941) was achieved by hsa-miR-122-5p, followed by AUC scores of 0.936, 0.864, 0.848, 0.843 and 0.738 for hsa-miR-30b-5p, hsa-let-7a-5p, hsa-miR-9-3p, hsa-miR-103-3p and hsa-miR-335-5p, respectively (Fig. 4).

Area under curve (AUC) was calculated for individual miRNAs in all infertile sub-groups as well. In oligo/oligoasthenozoospermic patients, the highest AUC (0.833) score was attained by hsa-miR-122-5p, followed by AUC scores of 0.819, 0.75, 0.696, 0.639, 0.585 and 0.556 for hsa-miR-30b-5p, hsa-miR-9-3p, hsa-miR-103-3p, hsa-let-7a-5p, hsa-miR-22-5p and hsa-miR-335-5p, respectively (Fig. 4). Thus, out of the seven differentially expressed miRNAs, three (hsa-miR-9-3p, hsa-miR-30b-5p, and hsa-miR-122-5p) had AUC above 0.75.

In case of normozoospermic infertile patients, the highest AUC (1.00) score was attained by hsa-miR-30b-5p, followed by AUC score of 0.997, 0.994, 0.938, 0.93, 0.90 for hsa-miR-122-5p, hsa-let-7a-5p, hsa-miR-103-3p, hsa-miR-335-5p and hsa-miR-9-3p, respectively (Fig. 4). Thus, all six differentially expressed miRNAs showed AUC above 0.75 in this group.

In asthenozoospermic patients, the highest AUC (1.00) score was attained by hsa-miR-122-5p and hsa-miR-30b-5p, followed by AUC score of 0.97, 0.90, and 0.90 for hsa-let-7a-5p, hsa-miR-9-3p and hsa-miR-103-3p, respectively (Fig. 4). Thus, all five differentially expressed miRNAs showed AUC above 0.75 in this group.

Five miRNAs (hsa-let-7a-5p, hsa-miR-9-3p, hsa-miR-30b-5p, hsa-miR-103-3p and hsa-miR-122-5p) showed AUC > 0.75 for infertile samples, three miRNAs (hsa-miR-9-3p, hsa-miR-30b-5p, and hsa-miR-122-5p) showed consistently high AUC across the three specific infertility categories, making them excellent markers of sperm quality. These three miRNAs were subjected to validation of their correlation with infertility by analysis in an independent set of samples.

### Validation of potential sperm quality markers

**Fig. 5:**
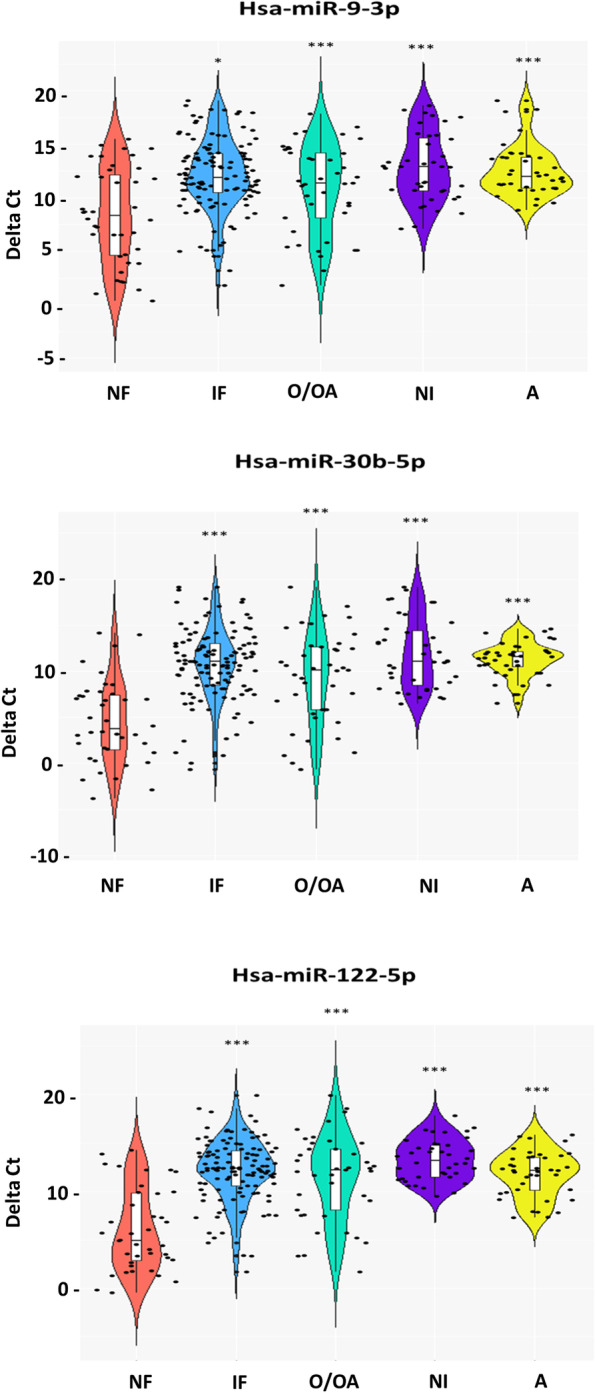
Violin plot showing the cumulative abundance levels of the three selected miRNAs. Non-parametric Mann–Whitney U test were used to evaluate differences in the abundance levels. Significant differences from the normozoospermic fertile control are indicated: *p < 0.05, **p < 0.005, ***p < 0.0005. NF, IF, O/OA, NI, A indicates normozoospermic fertile, infertile, oligo/oligoasthenozoospermic, normozoospermic infertile, asthenozoospermic groups, respectively

The three best miRNA candidates were further analysed in an independent set of normozoospermic infertile (*N* = 20), oligo/oligoasthenozoospermic infertile (*N* = 20), asthenozoospermic infertile (*N* = 20) and normozoospermic fertile (*N* = 20) semen samples. Cumulative analysis across fertile versus infertile categories showed significant down-regulations of all three in the infertile group as compared to the fertile group (Fig. 5). The mean abundance level was 0.07 –folds for hsa-miR-9-3p, 0.01 –folds for hsa-miR-30b-5p and 0.01 –folds for hsa-miR-122-5p in infertile samples as compared to fertile samples. Statistical comparison using Mann Whitney U test showed significantly reduced expressions of hsa-miR-9-3p (cases median ∆Ct 11.91, controls median ∆Ct 8.33), hsa-miR-30b-5p (cases median ∆Ct 11.04, controls median ∆Ct 3.74) and hsa-miR-122-5p (cases median ∆Ct 12.38, controls median ∆Ct 4.93) in the cases in comparison to the controls.

Across infertility sub-groups, all the three miRNAs (hsa-miR-9-3p, hsa-miR-30b-5p and hsa-miR-122-5p) were down-regulated in the oligo/oligoasthenozoospermic group in comparison to the normozoospermic group (Fig. 5). The mean abundance level was 0.17 –folds for hsa-miR-9-3p, 0.03 –folds for miR-30b-5p and 0.02 –folds for miR-122-5p in oligo/oligoasthenozoospermic samples as compared to normozoospermic fertile samples. Statistical comparison using Mann Whitney U test showed significantly reduced expressions of hsa-miR-9-3p (cases median ∆Ct 11.39, controls median ∆Ct 8.33), hsa-miR-30b-5p (cases median ∆Ct 10.11, controls median ∆Ct 3.74) and hsa-miR-122-5p (cases median ∆Ct 12.25, controls median ∆Ct 4.93) in the cases in comparison to the controls.

In the normozoospermic infertile group, all the three miRNAs (hsa-miR-9-3p, hsa-miR-30b-5p and hsa-miR-122-5p) were down-regulated in comparison to the normozoospermic fertile group (Fig. 5). The mean abundance level was 0.04 –folds for hsa-miR-9-3p, 0.006 –folds for hsa-miR-30b-5p and 0.006 –folds for hsa-miR-122-5p in normozoopsermic infertile as compared to normozoopsermic fertile samples. Statistical comparison using Mann Whitney U test showed significantly reduced expressions of hsa-miR-9-3p (cases median ∆Ct 12.90, controls median ∆Ct 8.33), hsa-miR-30b-5p (cases median ∆Ct 11.07, controls median ∆Ct 3.74) and hsa-miR-122-5p (cases median ∆Ct 13.19, controls median ∆Ct 4.93) in the cases in comparison to the controls.

In the asthenozoospermic infertile group, all the three miRNAs (hsa-miR-9-3p, hsa-miR-30b-5p and hsa-miR-122-5p) were down-regulated (Fig. 5). The mean abundance level was 0.05 –fold for hsa-miR-9-3p, 0.009 –folds for hsa-miR-30b-5p and 0.02 –folds for hsa-miR-122-5p in asthenozoopsermic infertile samples as compared to normozoospermic fertile samples. Statistical comparison using Mann Whitney U test showed significantly reduced expressions of hsa-miR-9-3p (cases median ∆Ct 11.98, controls median ∆Ct 8.33), hsa-miR-30b-5p (cases median ∆Ct 11.60, controls median ∆Ct 3.74) and hsa-miR-122-5p (cases median ∆Ct 12.17, controls median ∆Ct 4.93) in the cases in comparison to the controls.

### Quantitative (meta) analysis

**Fig. 6:**
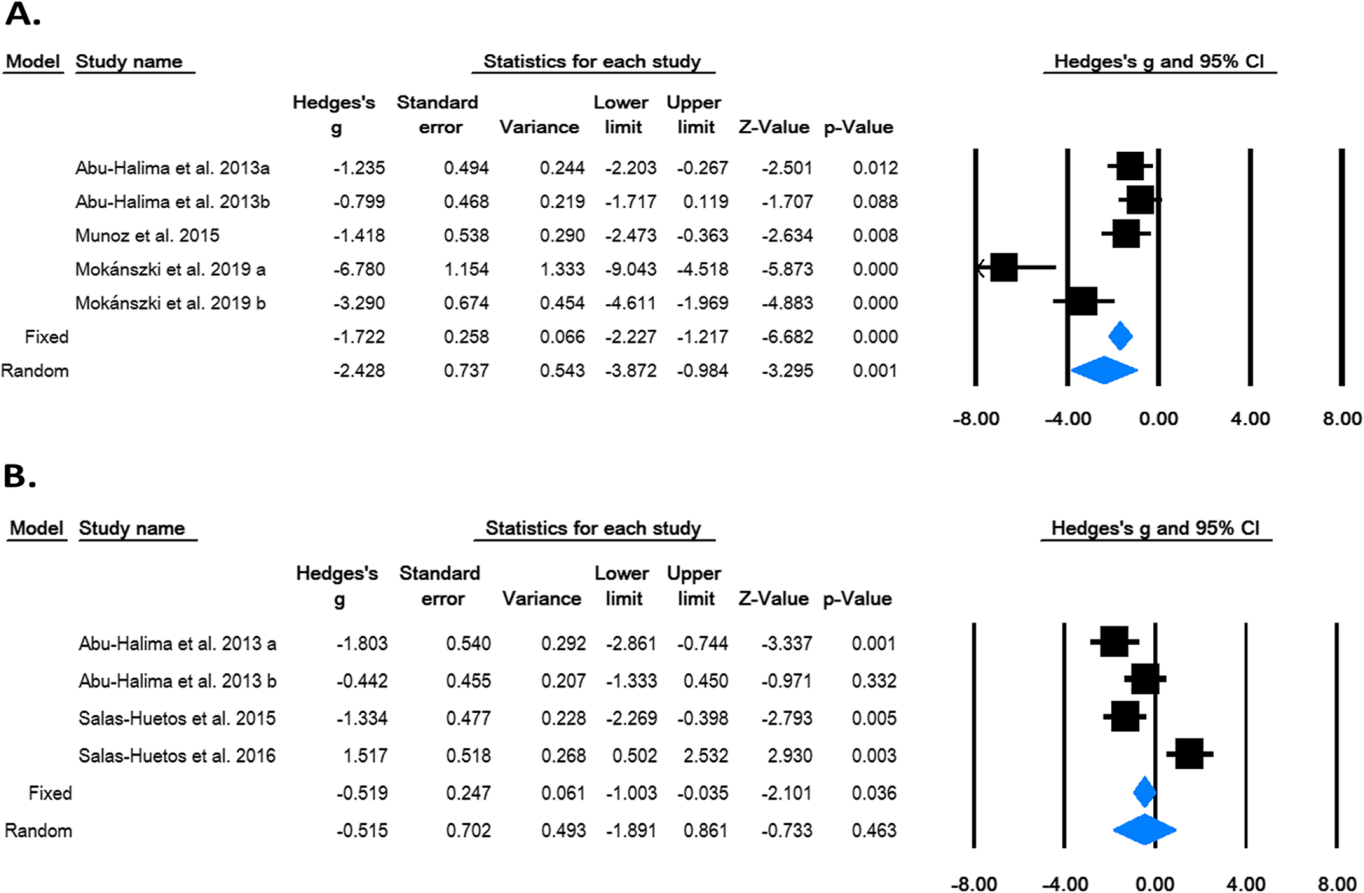
Forest plot for meta-analysis on relative expression of miR-122a-5p (**A**) and miR-9-3p (**B**). The Z value shows the degree and direction of relationship, whereas the p-value shows the significance of the relationship. The horizontal bar shows the range of mean with a square in the centre. The direction of the projection of the horizontal bar shows the direction of the association. The diamond-shaped box shows the pooled effect size and its width indicates the 95% CI (confidence interval)

We considered undertaking meta-analysis for microRNAs wherever three or more studies/data sets were available. Two miRNAs, hsa-miR-122-5p and hsa-miR-9-3p, qualified for quantitative meta-analysis. For quantification of the effect size, the input data taken were mean, SD and sample size. We chose the random effects model for interpretation because of high heterogeneity across studies (I^2^ = 86.57%, *p* = 0.000). The pooled analysis showed that hsa-miR-122-5p was significantly down-regulated in infertile men as compared to controls (Hedge’s g = -2.428, *p* = 0.001 (Random) (Fig. 6A). Egger’s regression intercept test suggested the existence of publication bias (intercept = -9.08, two-tailed *p*-value = 0.002).

In case of hsa-miR-9-3p, input data taken were mean, sample size and p-value. The pooled data showed high heterogeneity (I^2^ = 87.52%, *p* = 0.000). Pooled analysis showed that the expression level of hsa-miR-9-3p was significantly down-regulated in infertile men as compared to fertile controls in case of fixed model; however, the difference in the random effects model was not significant [(Hedge’s g = -0.519, *p* = 0.036 (Fixed) and Hedge’s g = -0.515, *p* = 0.463 (Random)] (Fig. 6B). The presence of publication bias was ruled out by Egger’s regression intercept test (intercept = 1.193, two-tailed *p*-value = 0.97).

### Target Prediction and functional analysis

**Fig. 7:**
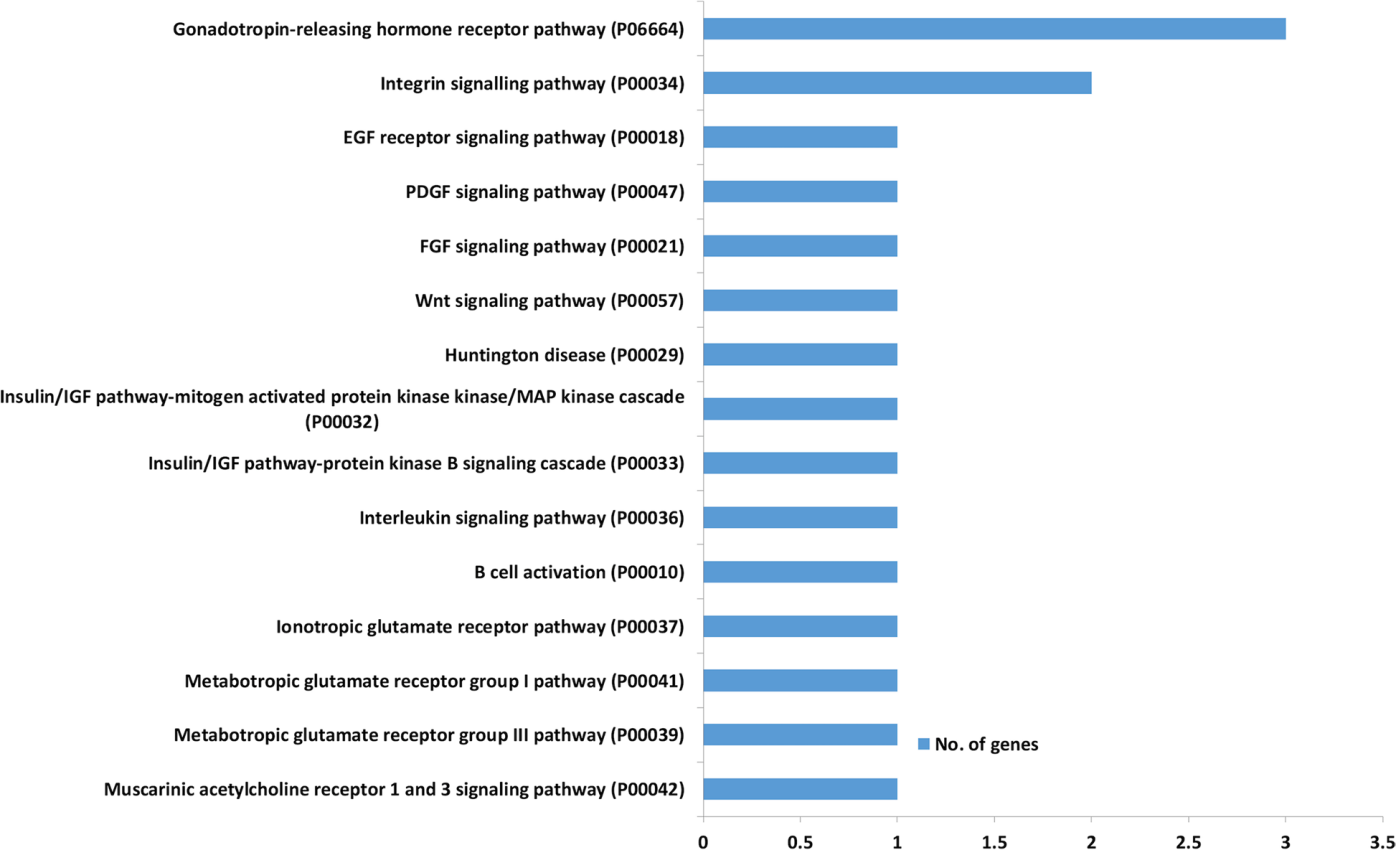
Pathway analysis for targets common to the three miRNAs, i.e. hsa-miR-9-3p, hsa-miR-30b-5p and hsa-miR-122-5p

Dysregulation of one miRNA expression in testes would alter the expression of a number of their target genes. The three most important miRNAs were looked for their targets, showing 1680, 2143 and 974 targets for hsa-miR-9-3p, hsa-miR-30b-5p and hsa-miR-122a-5p, respectively. Gene ontology of these targets showed their relation to metabolic processes, cellular processes, biological regulation, signalling, response to stimulus, developmental processes, multicellular organismal processes, and reproduction processes. In reproduction, these targets were related to meiotic cell cycle process, meiotic cell cycle, multi-organism reproductive processes, multicellular organismal reproductive processes, sperm motility, and developmental processes involved in reproduction. In molecular functions, the targets were related to binding, catalytic activity, molecular function regulation, transporter activity, and molecular transducer activity. In cellular processes, the targets were related to cellular anatomical entity, protein-containing complex and intracellular complex. Furthermore, pathway analysis for the targets common to the three miRNAs showed their association with signalling pathways like Wnt signalling pathway, gonadotropin-releasing hormone receptor pathway, EGF receptor signalling pathway, PDGF signalling pathway, FGF signalling pathway, and integrin signalling pathway (Fig. 7).

## Discussion

Among the earlier investigations on sperm/seminal miRNAs, Liu et al. (2012) undertook miRNA microarray on semen samples from 86 infertile males (mainly asthenozoospermic) and 86 normozoospermic fertile males and found 52 out of 2844 miRNAs to be differentially expressed [23]. Similarly, an year later, Abu-Halima et al. (2013) undertook microarray on 27 oligoastheno/asthenozoospermic sperm samples, analysing a total of 1205 human miRNAs [24]. Abhari et al. (2015) analysed only two miRNAs in 43 oligozoospermic infertile and 43 normozoospermic fertile sperm samples using qRT-PCR [25]. Similarly, Munoz et al. (2015) used qRT-PCR to analyse 23 miRNAs in sperm samples of 9 oligozoospermic infertile and 7 normozoospermic fertile males [27]. In the same year, Salas-Huetos et al. analysed 736 miRNAs by qRT-PCR in 10 oligozoospermic, 10 asthenozoospermic, 10 teretozoospermic infertile and 10 normozoospermic fertile sperm samples [26]. An year later, the same group analysed 736 miRNAs in sperm of 8 normozoospermic infertile and 8 normozoospermic fertile males by qRT-PCR [28]. Tian et al. (2018) performed qRT-PCR and analysed 6 miRNAs in semen of 36 oligozoospermic, 36 asthenozoopsermic, 36 oligoasthenozoopsermic infertile and 36 normozoospermic fertile males [29]. Recently, Mokánszki et al. (2020) analysed 11 miRNAs in both sperm and seminal plasma of infertile (oligozoospermic and asthenozoospermic) and normozoospermic fertile samples by qRT-PCR [30]. The above studies were sporadic and discrete efforts aimed at the identification of differentially expressed miRNAs in infertile sperm samples, which laid the foundation for advanced cross-studies analyses.

The evidence of abundance and heterogeneity of miRNAs in sperm and their significant differential expression in infertility prompted the studies on their potential utility as biomarkers of fertility or sperm quality. In the first ever such attempt, Abu-Halima et al. (2014) selected five miRNAs (hsa-miR-34b*, hsa-miR-34b, hsa-miR-34c-5p, hsa-miR-429, and hsa-miR-122) from their previous study [24, 35], ROC analysis on which suggested hsa-miR-34b*, hsa-miR-34b, hsa-miR-34c-5p, hsa-miR-122 and hsa-miR-429 to be good markers of fertility [22]. In another similar attempt, Corral-Vazquez et al. (2019) selected the best miRNA pairs from their previously published data having biomarker potential [26, 28, 36] and validated their expression in nine fertile and fourteen infertile sperm samples. The results showed that the hsa-miR-942-5p/hsa-miR-1208 pair displayed the best potential for the diagnosis of infertile men with seminal alterations while the hsa-miR-34b-3p/hsa-miR-93-3p pair showed the best potential for the diagnosis of infertile men with unexplained infertility or when seminal parameters were close to the threshold values [2]. Thus, the initial evidence had suggested the potential utility of sperm miRNAs as fertility markers; however, a global search across all published data in the last one decade had never been undertaken.

On comparison of miRNA differential expression data across all published studies, including our case–control sequencing data [31], we found 497 differentially expressed miRNAs (DE-miRNAs) in sperm/semen of infertile patients as compared to fertile controls. Consistent differential expression of seven miRNAs across diverse studies indicated their strong association with infertility, suggesting their potential as markers of sperm fertility/quality. We analysed these seven miRNAs in a set of fertile and infertile sperm samples to consolidate on their association with infertility and their suitability as biomarkers of sperm fertility/quality. While six out of seven miRNAs showed correlation with infertility, four (hsa-miR-9-3p, hsa-miR-30b-5p, hsa-miR-103-3p and hsa-miR-122-5p) miRNAs were consistently differentially expressed across all sub-groups, suggesting their strong linkage with infertility. ROC curve analysis on these suggested hsa-miR-9-3p, hsa-miR-30b-5p, and hsa-miR-122-5p to be the best molecular biomarkers of sperm quality. We expanded the analyses of these three promising miRNAs in another set of sperm samples, finding their significant down-regulation in infertility. The linkage of hsa-miR-122-5p with infertility was also strengthened by meta-analysis, while hsa-miR-9-3p showed significant association in the fixed effects model of meta-analysis. Adequate quantitative data on hsa-miR-30b-5p was not available for undertaking a meta-analysis. Further studies on sperm miRNAs would identify more miRNAs as potential biomarkers of sperm fertility/quality.

Hsa-miR-9-3p, hsa-miR-30b-5p and hsa-miR-122-5p have been reported to be abundantly and consistently expressed in testis and sperm of fertile males, suggesting their suitability as biomarkers [37]. Dysregulations of these three miRNAs in testis and sperm have been associated with germ cells arrest and abnormalities in sperm formation [37], suggesting their biological significance in spermatogenesis. However, only miR-122 has been molecularly investigated for its role in spermatogenesis; it regulates the expression of TNP2 gene, which is required for proper compaction of chromatin during spermiogenesis [38]. Interestingly, all three miRNAs have been reported to play crucial roles in human embryo development [39–41]. Sperm miRNAs may be critical during spermatogenesis or post-fertilization development or may have dual functions. Our findings not only point out their significance as sperm quality biomarkers, but also as excellent candidates for investigation of their roles in spermatogenesis. Target prediction showed enrichment of the Wnt signalling pathway, gonadotrophin releasing hormone receptor pathway, EGF receptor signalling pathway, PDGF signalling pathway, FGF signalling pathway and integrin signalling pathway, a number of which play critical roles in spermatogenesis and post-fertilization development [42–46]. Among other miRNAs out of seven interrogated in this study, hsa-let-7a-5p, hsa-miR-103-3p, are also worth investigating for their role in spermatogenesis as they have been correlated with post-fertilization development or embryo quality [41].

## Conclusions

In conclusion, our systematic investigation on published data identified a number of potential miRNA markers of infertility, of which hsa-miR-9-3p, hsa-miR-30b-5p and hsa-miR-122-5p showed consistent and rigorous association with infertility. These miRNAs could be good biomarkers for assessing sperm quality and may be worth adopting in ART clinics. These miRNAs can also be used for RNA therapeutics of infertility using ART in the cases with sperm molecular abnormalities with or without alterations in the semen profile. Oligo/oligoasthenozoospermia category showed maximum variations in the level of expression in comparison to other categories. Further validation of these miRNAs in other populations would strengthen their clinical utility. Sharing of 21 miRNAs across three studies and 80 miRNAs across two studies suggests the existence of a number of other potential biomarkers, which can be subjected to similar investigations for their association with infertility. High cost of analysis, critical role of RNA preparation quality and relatively narrow range of variation in their expression are some of the limitations of using miRNAs as biomarkers of infertility. It is noteworthy that all studies on sperm miRNA in infertility, except our study [31], were microarray or RT-PCR based, next generation sequencing based studies would identify as yet unidentified miRNAs in sperm.

## Supplementary Information

**Additional file 1: Supplementary Table 1**. Correlation of miRNA abundance level (∆Ct) and semen parameters.

## Data Availability

All the data were available upon the request from the corresponding authors.

## Abbreviations

ROS: Reactive oxygen species
IVF: In vitro fertilization
ROC: Receiver operating characteristic
AUC: Area under ROC curve
ART: Assisted reproductive technique
L-GQE: Low rate of good quality embryos
H-GQE: High rate of good quality embryos
EER: Effective embryo rate
HQER: High quality embryo rate
