# Supplementary material for: Qualitative and quantitative assessment of sperm miRNAs identifies hsa-miR-9-3p, hsa-miR-30b-5p and hsa-miR-122-5p as potential biomarkers of male infertility and sperm quality
Source: Reprod Biol Endocrinol. 2022 Aug 15;20:122. doi: 10.1186/s12958-022-00990-7 (PMC9377062; doi:10.1186/s12958-022-00990-7)
Supplement: Supplementary file 1 — Additional file 1: Supplementary Table 1. Correlation of miRNA abundance level (∆Ct) and semen parameters. [file 12958_2022_990_MOESM1_ESM.docx]

**Supplementary Table 1:** Correlation of miRNA abundance level (∆Ct) and semen parameters.

| **Sperm concentration (10^6^ / ml)** | | | | | | | | | | | | |
| --- | --- | --- | --- | --- | --- | --- | --- | --- | --- | --- | --- | --- |
| Parameters | miR-122-5p | | miR-30b-5p | | miR-9-3p | miR-103-3p | | Let-7a-5p | | miR-22-5p | | miR-335-5p |
| r | -0.438 | | -0.450 | | -0.312 | -0.123 | | -0.088 | | 0.069 | | 0.015 |
| 95% confidence interval | -0.6627 to -0.1420 | | -0.6710 to -0.1566 | | -0.572 to 00035 | -0.4193 to 0.1968 | | -0.3976 to 0.2380 | | -0.2517 to 0.3778 | | -0.3023 to 0.3299 |
| P value | 0.004 | | 0.003 | | 0.046 | 0.437 | | 0.586 | | 0.664 | | 0.924 |
| **Sperm motility (%)** | | | | | | | | | | | | |
| Parameters | | miR-122-5p | | miR-30b-5p | | | miR-9-3p | | miR-103-3p | | Let-7a-5p | |
| r | | -0.795 | | -0.771 | | | -0.736 | | -0.559 | | -0.786 | |
| 95%  confidence interval | | -0.8908 to -0.6314 | | -0.8778 to -0.5940 | | | -0.8578 to -0.5389 | | -0.7542 to -0.2729 | | -0.8892 to -0.6075 | |
| P value | | <0.001 | | <0.001 | | | <0.001 | | <0.001 | | <0.001 | |
